# Gout on the acute medical take

**DOI:** 10.1016/j.clinme.2025.100331

**Published:** 2025-05-26

**Authors:** Abhishek Abhishek, Edoardo Cipolletta

**Affiliations:** a Academic Rheumatology, City Hospital Nottingham, University of Nottingham, Nottingham, NG5 1 PB, UK; b Rheumatology Unit, Department of Clinical and Molecular Sciences, Polytechnic University of Marche, Ancona, 60126, Italy

**Keywords:** Gout, Synovial fluid, Ultrasonography, Dual energy CT, NSAIDS, Colchicine, Glucocorticoids

## Abstract

Gout is the commonest form of inflammatory arthritis. Flares are the commonest presentation of gout. Typically, gout flares present as acute monoarthritis, and most often affect the first metatarsophalangeal joint (‘*podagra*’). Other joints in the lower limbs are affected more often than those in the upper limbs. Joint aspiration followed by examination of the synovial fluid using a polarised light microscope is the gold standard for a definite diagnosis of gout. Gout may be diagnosed without recourse to joint aspiration if there is podagra, elevated serum urate and no suspicion of infection. Ultrasonography and dual energy computed tomography may be used to diagnose gout if joint aspiration is unsuccessful or not feasible. Oral colchicine, NSAIDs and glucocorticoids have similar efficacy for controlling gout flare with differing adverse effect profiles. Consequently, the drug choice depends on comorbidities and patient preference.

## Highlights

• Synovial fluid aspiration and prompt examination of the aspirated synovial fluid under polarised light microscopy are central to definite diagnosis of gout.
• Ultrasonography and dual energy computerised tomography may be used if joint aspiration is not feasible or unsuccessful.
• Oral colchicine, NSAIDs, and glucocorticoids have similar efficacy for controlling gout flare with different side effect profile.
• Drug choice depends on comorbidities and patient preferences.
• Healthcare contact for gout flares provides an opportunity to discuss and otimise urate-lowering treatment.

## Introduction

Gout results from hyperuricaemia that causes the deposition of monosodium urate (MSU) crystals in articular and periarticular tissues. MSU crystals cause inflammation by stimulating the NLRP-3 (NACHT, leucine-rich region, and PYD domains containing protein 3) inflammasome that activates pro-interleukin(IL)1β.^1^ Contrary to previous beliefs that gout is caused by dietary and lifestyle excesses alone, recent evidence shows that genetically driven renal and gastrointestinal underexcretion of urate is a major contributor to the pathogenesis of hyperuricaemia and gout.^2^^,^^3^ Gout affects 2.5% adults in the UK and its prevalence increases with increasing age.^4^ It affects men more often than women.^4^ Women generally develop gout after the menopause due to the absence of uricosuric effect of oestrogen in the postmenopausal period.^5^ Gout is associated with an increased risk of premature mortality and with comorbidities such as obesity, hypertension, hyperlipidaemia, cardiovascular disease and chronic kidney disease.^6^^,^^7^

### Clinical presentations of gout

*Gout flare:* Gout flare is often the first manifestation. It is characterised by an abrupt onset of severe joint pain that commonly begins overnight or in the early hours of the morning, with swelling, warmth, and redness of the overlying skin.^8^ Flares often achieve peak pain intensity within 12–24 h. Most gout flares are monoarticular, but more than one joint can be affected. Fever and other features of systemic inflammation may be present. Flares typically resolve within a few days to 1–2 weeks. The first metatarsophalangeal joint is the commonest location of gout flare (podagra). Knees, ankles and the midfoot are also frequently affected, while joints in the upper limbs, eg elbows and wrists, are affected less often.^8^

**Fig. 1:**
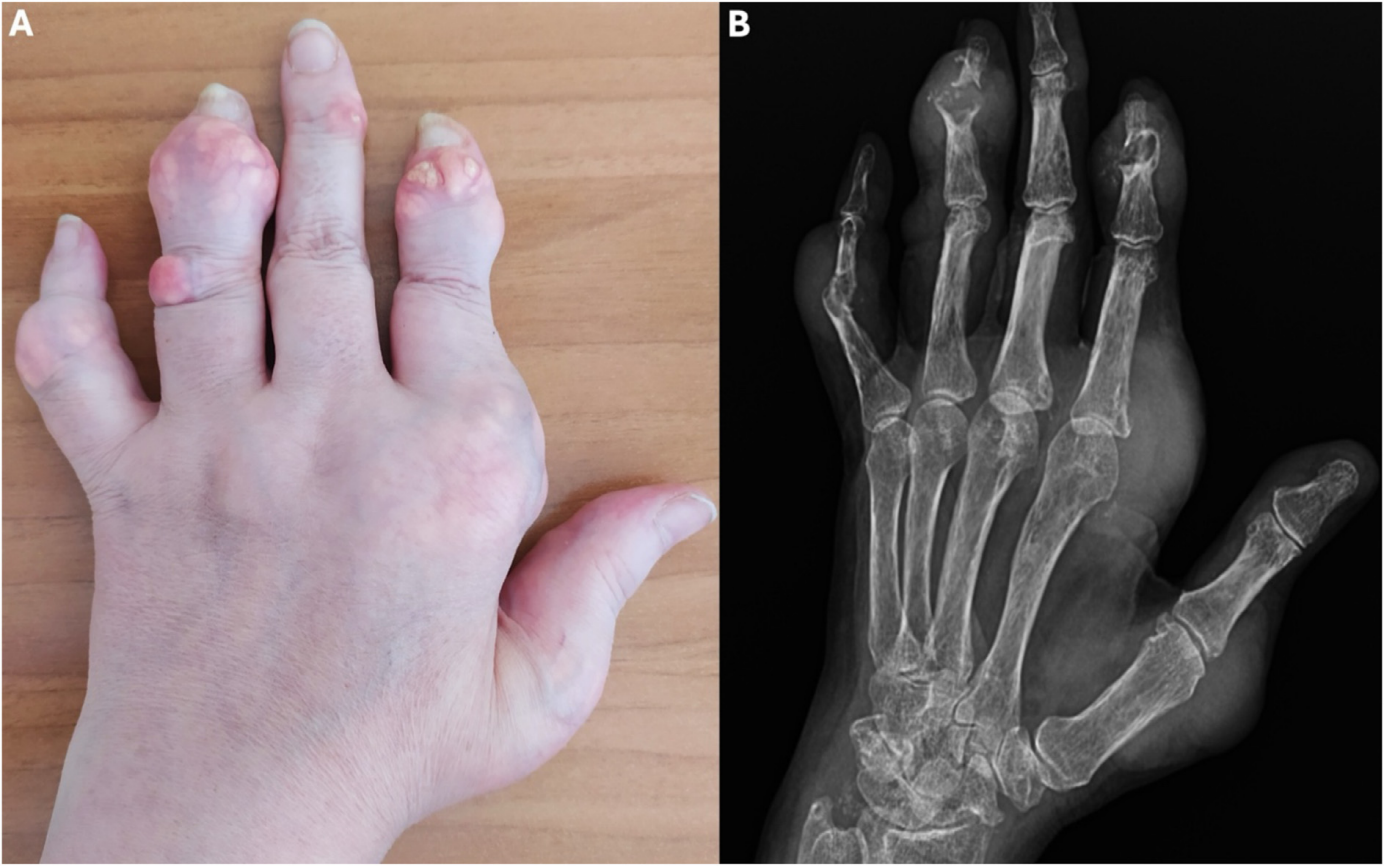
Tophaceous gout. Comparison of findings detectable on physical examination (Panel A) and conventional radiography (Panel B). Please note the yellow/white appearance of tophi in panel A and the extensive bone destruction around them (eg second and fourth distal interphalangeal joints) in panel B. Written permission was obtained from the patient for using the images in a publication.

*Tophaceous gout:* This is characterised by subcutaneous tophi, ie yellow or white hard subcutaneous nodules (Fig. 1A) that comprise MSU crystals and inflammatory and fibrotic tissue.^9^ There may be destructive arthropathy (Fig. 1B). Tophaceous gout usually occurs due to poorly treated disease over many years, but can be the first presentation in some, eg those with solid organ transplant.

### Approach to a suspected gout flare

Joint aspiration followed by prompt examination of the synovial fluid using a polarised light microscope is the gold standard for a definite diagnosis of gout.^10^ The presence of negatively birefringent needle-shaped MSU crystals is confirmatory. Gram stain and culture of the synovial fluid should be performed to exclude septic arthritis.

Gout may be diagnosed without recourse to joint aspiration if there are typical clinical features, ie rapid onset of joint pain with swelling and warmth of the first metatarsophalangeal joint (podagra) with elevated serum urate in a man or postmenopausal woman.^10^ Localised erythema may also be present. Joint aspiration should be performed for other clinical presentations or if there is a suspicion of septic arthritis. Ideally, this should be done before antibiotics are administered unless there is sepsis. In that case, the administration of intravenous antibiotics takes precedence.

Ultrasound guidance may be used in case the joint aspiration cannot be undertaken using bony landmarks or is unsuccessful.

**Role of imaging:** Plain radiography, ultrasonography, and dual energy computed tomography (DECT) may be used to diagnose gout.^11^ Plain radiography often reveals soft tissue swelling during gout flare – a non-specific finding. Infrequently, there may be typical juxta-articular punched-out erosions with preserved margins. As erosions are only present in advanced disease, plain radiography has low sensitivity for gout diagnosis.^12^

**Fig. 2:**
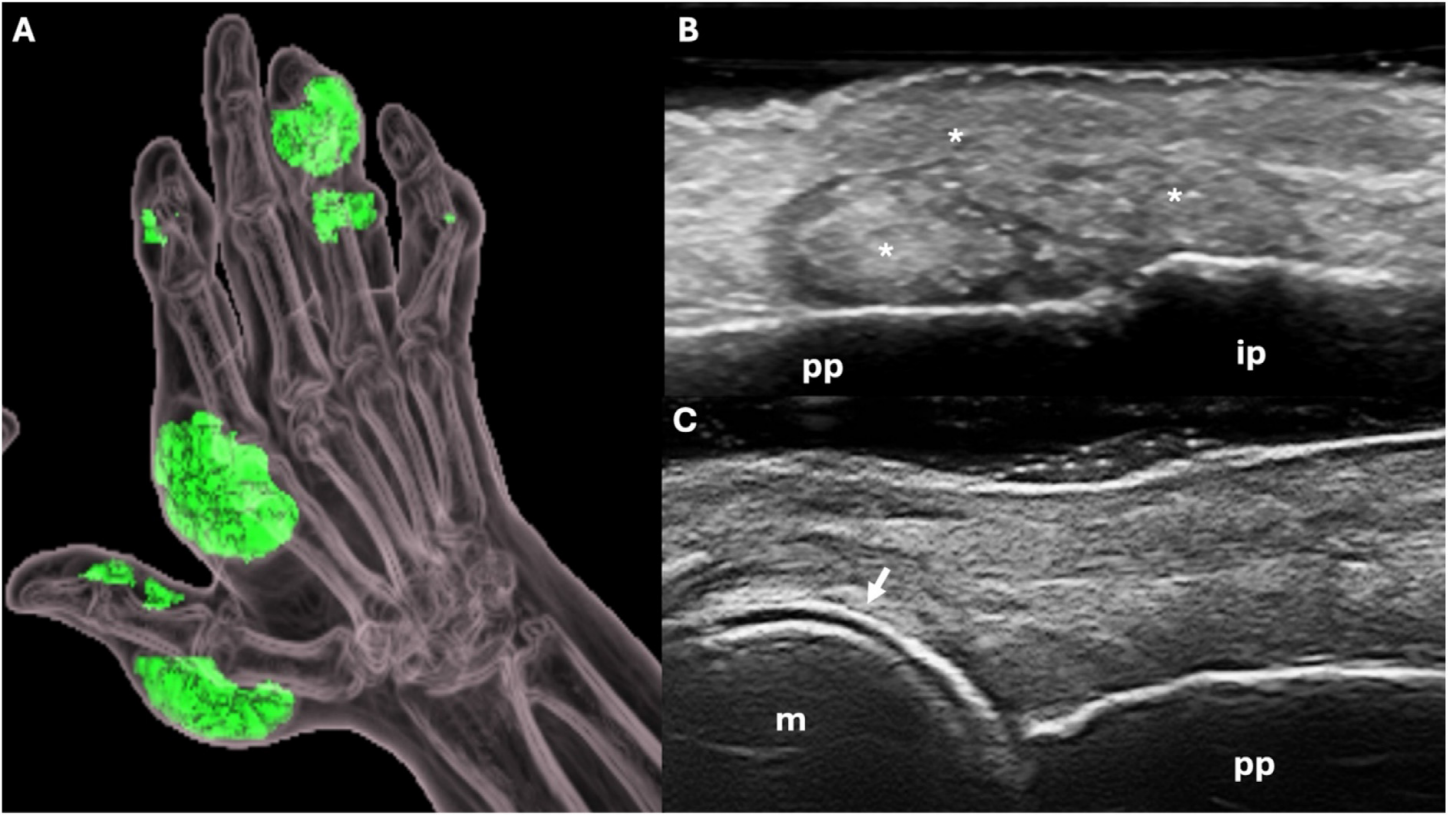
Panel A: dual energy CT image showing MSU crystal deposits in green in a patient with long-standing tophaceous gout. Panel B: long-axis view of the proximal interphalangeal joint of the fourth digit of the hand. Intra- and extra-articular MSU crystal deposits (asterisks: tophi, pp: proximal phalanx, ip: intermediate phalanx). Panel C: long-axis view of the metacarpophalangeal joint of the second digit of the hand. Double contour sign (arrow: double contour sign, m: metacarpal bone, pp: proximal phalanx). Written permission was obtained from the patient for using the images in a publication.

Ultrasonography and DECT have greater sensitivity for detecting MSU crystal deposits.^12^ Double contour sign or tophi on ultrasonography, and urate deposition on DECT are typical findings in gout (Fig. 2).

Blood tests undertaken during a gout flare may reveal neutrophilia, thrombocytosis and elevated CRP and/or ESR. The serum urate level may be normal due to transient uricosuria during acute inflammation (urate is a negative acute phase reactant).^13^ Therefore, a serum urate <360 µmol/L should not be used to exclude gout in people with acute arthritis. Similarly, serum urate ≥360 µmol/L is common and by itself does not suggest gout.

### Differential diagnosis of gout flare

Differential diagnoses of gout flare include septic arthritis, acute calcium pyrophosphate (CPP) crystal arthritis, calcific peri-arthritis, hemarthrosis, reactive arthritis, and psoriatic arthritis.

Differentiation between gout flare and either septic arthritis or acute CPP crystal arthritis (pseudogout) is challenging. The presence of gouty tophi can be a useful pointer. There may be risk factors for infection in those with septic arthritis. Acute CPP crystal arthritis typically affects those older than 60–70 years in age. Knees and wrists are the most affected joints, and the first metatarsophalangeal joint is affected extremely rarely. Results of synovial fluid analysis and blood culture are incredibly helpful in excluding these conditions. CPP crystals can be hard to visualise as they are smaller and either non-birefringent or only weakly positively birefringent. If the synovial fluid aspiration did not demonstrate CPP crystals, plain radiographs, ultrasonography and CT may be helpful in detecting CPP deposition (visualised as chondrocalcinosis). Plain radiography has low sensitivity for chondrocalcinosis.^12^ Ultrasonography has both high sensitivity and specificity for chondrocalcinosis.^12^

Calcific periarthritis due to basic calcium phosphate crystals presents with periarticular joint pain, swelling and tenderness, and typically affects the shoulders and the hip. There may be well-demarcated nummular soft-tissue calcification. A history of trauma and/or anticoagulation suggests haemarthrosis and can be confirmed using joint aspiration.

Reactive arthritis and psoriatic arthritis can present as acute mono- or oligoarthritis. A recent history of gastroenteritis (eg due to *Campylobacter, Salmonella, Shigella*) or sexually transmitted infection (eg due to *Chlamydia*) raises the possibility of reactive arthritis. A personal or family history of psoriasis in a first-degree relative and recent infection points towards the alternate diagnosis. Nevertheless, aspiration of the affected joint and examination of the synovial fluid aids certainty to the diagnosis.

### Management of gout flares

Rest, cold pack, paracetamol ± weak opioids should be used for symptom control.^10^ Anti-inflammatory drugs should be started as soon as possible. Low-dose aspirin and urate-lowering drugs, eg allopurinol, should not be discontinued during a gout flare.

Although there is no randomised controlled trial (RCT) evidence supporting their effectiveness, intra-articular glucocorticoid injections are often administered if there are one or two affected joints amenable to joint injection. The typical dose varies from 10 mg (interphalangeal joints) to 40 mg (knees, ankles, wrists) long-acting glucocorticoid (eg methylprednisolone or triamcinolone) administered in a single injection, depending on the affected joint. It is important to exclude septic arthritis before glucocorticoids are prescribed. While this delays treatment, it minimises risks associated with glucocorticoid exposure to an infected joint.

Oral glucocorticoids, colchicine and NSAIDs may be used to treat gout flares. Anakinra (anti-IL1) may be used if other drugs are contraindicated. All four have comparable efficacy, with differing contraindications and side-effect profile. Consequently, drug choice is governed by contraindications and patient preference (Table 1). Typically, a single agent is sufficient to control gout flare. Rarely, either glucocorticoids or NSAIDs may need to be combined with colchicine to control severe flares. It is advisable to not combine oral glucocorticoids and NSAIDs due to risk of gastrointestinal bleeding.

**Table 1:** Choice of anti-inflammatory drug therapy to treat gout flares.

	Colchicine^a^	Oral glucocorticoids	Intra-articular glucocorticoids	NSAIDs
Older age, eg >65 years	Caution^b^	Yes	Yes	Avoid
Chronic kidney disease (eGFR<60 mL/min)	Caution^b^	Yes	Yes	Avoid
Heart failure	Yes	Avoid	Caution	Avoid
Coronary heart disease	Yes	Yes	Yes	Avoid
Anticoagulated	Yes	Yes	No	Avoid
Hypertension	Yes	Caution	Yes	Avoid
Peptic ulcer/past gastrointestinal bleeding	Yes	Avoid	Yes	Avoid
Diabetes mellitus	Yes	Avoid	Caution	Yes
Suspected septic arthritis or systemic infections	Yes	No	No	Yes

a Colchicine has several drug interactions, and its dose may need to be reduced if there are interacting drugs co-prescribed.

b Reduce dose.

In RCTs, prednisolone 30 mg/day for 5 days had comparable efficacy, but better safety profile compared to indomethacin 150 mg/day for 2 days followed by 75 mg/day for 3 days;^14^ prednisolone 35 mg/day for 5 days had comparable efficacy and safety profile compared to Naproxen 1,000 mg/day for 5 days;^15^ and Naproxen 750 mg immediately followed by 250 mg every 8 hours for 7 days had comparable efficacy, but better safety profile with fewer instances of diarrhoea compared to colchicine 0.5 mg three times a day for 4 days.^16^ Loading dose of colchicine should be avoided due to a very high risk of diarrhoea.^17^ In another RCT, IL-1 inhibitor anakinra (100 mg/day subcutaneous injection for 5 days) had comparable efficacy to other anti-inflammatory agents in treating gout flare.^18^

*Long-term management:* Healthcare contact for gout flares is an opportunity to discuss urate-lowering treatment (ULT). Despite evidence that treat-to-target ULT prevents gout flares, gout management remains suboptimal in the UK, with most people with gout not prescribed any ULT and, where ULT is prescribed, the dose is not titrated to a serum urate treatment target.^19^ ULT, eg allopurinol 100 mg/day, may be commenced during a gout flare as there is no evidence that this approach would worsen gout flare severity in several RCTs.^20^ A lower starting dose of allopurinol should be used in renal impairment.^10^ If ULT is initiated during a gout flare, flare prophylaxis should be prescribed, and the dose of ULT should be up-titrated at 2–4-weekly intervals, guided by the latest serum urate level, until a serum urate <360 µmol/L (or <300 µmol/L in those with tophi) is achieved.^10^

Advice on improving diet and lifestyle when indicated, and assessment for comorbidities form part of gout flare assessment. Drugs that can cause hyperuricaemia, eg diuretics and beta-blockers, should be discontinued where possible and drugs like losartan and SGLT2-inhibitors that also have an uricosuric effect should be considered.

## Conclusion

Synovial fluid examination remains the cornerstone for diagnosing gout. Ultrasonography and DECT are useful when joint aspiration is unfeasible, unsuccessful or does not demonstrate MSU crystals. There are several effective treatments for gout flares. Drug choice should be individualised to comorbidities and patient preference.

## Funding

This research did not receive any specific grant from funding agencies in the public, commercial or not-for-profit sectors.

## CRediT authorship contribution statement

**Abhishek Abhishek:** Writing – review & editing, Writing – original draft, Visualization, Supervision, Software, Resources, Project administration, Methodology, Investigation, Conceptualization. **Edoardo Cipolletta:** Writing – review & editing, Writing – original draft, Methodology, Investigation, Conceptualization.

## Declaration of competing interest

The authors declare the following financial interests/personal relationships which may be considered as potential competing interests: Abhishek reports a relationship with Swedish Orphan Biovitrum AB publ that includes: speaking and lecture fees. Abhishek reports a relationship with UptoDate Inc that includes: speaking and lecture fees. Cipolletta reports a relationship with Horizon Therapeutics USA Inc that includes: speaking and lecture fees. Cipolletta reports a relationship with Novartis that includes: speaking and lecture fees. Cipolletta reports a relationship with IBSA Farmaceutici Italia Srl that includes: speaking and lecture fees. If there are other authors, they declare that they have no known competing financial interests or personal relationships that could have appeared to influence the work reported in this paper.
